# Diagnostic value of the gynecology imaging reporting and data system (GI-RADS) with the ovarian malignancy marker CA-125 in preoperative adnexal tumor assessment

**DOI:** 10.1186/s13048-018-0465-1

**Published:** 2018-11-03

**Authors:** Michal Migda, Migda Bartosz, Marian S. Migda, Marcin Kierszk, Gieryn Katarzyna, Marek Maleńczyk

**Affiliations:** 1 Clinical Unit of Obstetrics, Women’s Disease and Gynecological Oncology, sw. Jozefa 53/59, United District Hospital, Collegium Medicum University of Nicolaus Copernicus in Toruń, Torun, Poland; 2 Department of Diagnostic Imaging, Second Faculty of Medicine with the English Division and the Physiotherapy Division, Medical University of Warsaw, Warsaw, Poland; 3 Civis Vita Medical Center, Torun, Poland

**Keywords:** Gynecology imaging reporting and data system, Ca-125, Ultrasound, Ovarian tumor

## Abstract

**Objectives:**

The purpose of this study is to assess the preoperative evaluation of an adnexal mass using the GI-RADS classification and to verify whether CA-125 measurement can offer any additional benefits to the GI-RADS-based prediction of ovarian tumor malignancy.

**Material and methods:**

In this study, we assessed a total of 215 women with an adnexal tumor using the GI-RADS classification combined with CA-125 measurement. All adnexal masses underwent histological verification.

**Results:**

Of a total of 215 lesions, we classified 2 lesions as GI-RADS 2 (0.9%), 118 lesions as GI-RADS 3 (54.9%), 86 lesions as GI-RADS 4 (40.0%) and 9 lesions as GI-RADS 5 (4.2%). For GI-RADS 4–5 lesions, the sensitivity, specificity, PPV, NPV, ACC and OR were as follows: 94.3, 72.2, 52.6, 97.5, 77.7%, and 43.3 (CI 12.0–146), respectively. The corresponding parameters resulting from combining the GI-RADS classification with the CA-125 marker were as follows: 66.0, 93.8, 77.8, 89.4, 87.0%, and 29.6 (CI 12.6–69.6), respectively, with *p* < 0.001. For Ca-125 > 30 IU/mL alone, the results were as follows: 70.0, 80.3, 53.8, 89.1, 77.7%, and 9.5 (4.6–19.6), respectively, with *p* < 0.0001.

Additionally, 47.8% of the patients had no symptoms, 36.5% had back pain, 5.2% had an increased abdominal size, 4.3% had menstrual irregularities and 2.6% had constipation. There were 152 benign and 18 malignant cases in the low risk group (GIRADS 1–3 and GIRADS 4 + CA-125 < 30 IU/mL) and 10 benign and 35 malignant tumors in the high-risk group (GIRADS 4 + CA125 > 30 IU/mL and GIRADS 5).

**Conclusions:**

GI-RADS classification had good performance in discriminating ovarian tumors. The additional measurement of CA-125 improves the system specificity, PPV and ACC for preoperative adnexal tumor assessment.

## Introduction

Ovarian cancer is the most lethal cancer among gynecological malignancies. It has been estimated that over 151,000 women died from this disease in 2012 worldwide [1]. In Poland, ovarian cancer is the second most frequently diagnosed malignancy of the female genital tract, with an incidence rate of 3600 new cases per year, and has the highest mortality among gynecological cancers, reaching 2600 deaths every year [2]. Sadly, nearly 70% of patients with ovarian cancer are diagnosed at an advanced stage, while the 5-year survival rate for patients with ovarian cancer may be as high as 90% when treated early [3]. It has been demonstrated that the survival of ovarian cancer patients is better when treatment is provided at specialized centers by gynecologists with expertise in gynecologic oncology [4]. To date, surgical treatment, chemotherapy, radiotherapy, biotargeted therapy and other technologies have improved. Screening tumor markers using gene chip technology by detecting the hypomethylation of certain genes may be potentially helpful in high-risk groups, such as BRCA1 and BRCA2 patients, but not in the general population [5]. Studies based on proteomics are based on appropriate protein analysis technology such as surface-enhanced laser desorption/ionization time-of-flight (TOF)-MS, which shows 100% sensitivity and 93.3% specificity, indicating that this approach is useful for diagnosing ovarian cancer [6]. A cytogenetic analysis study by Lagana showed that the progression of epithelial ovarian cancer is characterized by a series of combined epigenetic aberrations determined by loss of methylation of certain regions of DNA encoding genes such as the Ras-association domain-containing family 1 (RASSF1A) tumor suppressor, which is considered a new diagnostic development [7]. Additionally, technical improvement allows surgery on a patient with an early stage of ovarian carcinoma using laparoscopy or robot-assisted laparoscopy, making this an acceptable approach for this selected group [8]. The preoperative assessment of an adnexal mass is difficult, which leads to a disproportionate number of women with benign ovarian tumors being referred to specialized centers and, conversely, women with ovarian malignancy being inappropriately operated on in nonspecialized centers [4]. Ultrasonography is currently considered the primary imaging modality for identifying and characterizing adnexal masses [9]. Due to the subjective nature of the examination, there has been a need for standardized nomenclature and a definition of all tumor features evaluated by ultrasound. The International Ovarian Tumor Analysis provides consensus on ultrasonography nomenclature and definitions of all tumor features and has improved the discrimination of adnexal masses by including a quantitative assessment of some morphological features [10]. In 2009, based on the Breast Imaging Reporting and Data System (BI-RADS), Amor et al. proposed a Gynecology Imaging Reporting and Data System (GI-RADS) as a similar system to facilitate communication between sonographers and referring clinicians [11]. The contemporary diagnostic standard for ovarian cancer includes transvaginal ultrasound and the measurement of serum CA-125. A wide range of other diagnostic approaches is being investigated at present [1].

The purpose of this study was to assess the performance of the GI-RADS reporting system in the preoperative discrimination of adnexal masses in Polish women and to test whether the measurement of CA-125 can offer any additional benefits to the GI-RADS risk evaluation for the malignancy of ovarian tumors.

## Materials and methods

This study was approved by the board of Clinical Unit of Obstetrics, Women’s Disease and Gynecological Oncology, United District Hospital, Collegium Medicum University of Nicolaus Copernicus in Toruń, Poland. Over a 24-month period, we enrolled a total of 215 women with adnexal masses into the study. The inclusion criteria were primarily based on the clinical diagnosis of an adnexal mass followed by ultrasound confirmation at our tertiary center and the obtaining data indicating pathology. Patients with pregnancy, bilateral adnexal tumors or a malignancy diagnosis already established were excluded from the study.

Patients were assessed by an experienced examiner (500 scans a year) 2–3 days prior to surgery. Vaginal and transabdominal two-dimensional (2D) ultrasound examinations were performed using a Voluson E8 (GE Medical Systems, Zipf, Austria). Morphological features were examined according to GI-RADS and included unilateral involvement, the maximum diameter of the lesion, the wall thickness, septa, solid papillary projections, solid areas within the cyst, cystic content and ascites [6]. Color Doppler was used to assess peripheral or central vascularization.

Peripheral blood was collected for the measurement of serum CA-125 1 to 14 days prior to surgery. Blood was collected from all patients and stored in serum separator tubes. Automated analysis of CA-125 was performed by direct chemiluminescence using an Advia Centaur CA-125 II assay (Siemens Medical Solutions Diagnostics, Tarrytown, USA). Values were expressed in international units per milliliter (IU/mL).

A definitive histological diagnosis was obtained from surgical excision or a biopsy sample. Tumors were classified according to the WHO criteria [8]. Borderline tumors were considered malignant for the purposes of the present study. Statistical analysis was performed using the statistical software STATISTICA 10 (StatSoft Inc.). GI-RADS classification was combined with a CA-125 assay, and descriptive measures were calculated (for CA-125 > 30 IU/mL): sensitivity, specificity, positive predictive value (PPV), negative predictive value (NPV), accuracy (ACC), and odds ratio (OR) at a 95% confidence interval. In all cases of a categorical variable comparison, a Chi-squared test was used. In the case of GI-RADS, categories 2 and 3 were considered low-risk, while categories 4 and 5 were considered high-risk. Histological diagnosis was used as a gold standard. Continuous variables, such as age, were assessed using a Mann-Whitney U test. For all analyses, *p* < 0.05 was considered significant.

## Results

The study was based on the analysis of 215 unilateral adnexal tumors. The average age of the patients was 47.2 years old (range = 13–89). The average age of the patients in the malignant tumor group was significantly higher than that in the benign tumor group: 60 years (range 36–89) vs 43.1 years (range 13–84), respectively, with a *p*-value of < 0.001 for both groups. We found a total of 53 masses to be malignant (24.7% of all adnexal tumors). In the 215 tumors, 2 lesions were classified as GI-RADS 2 (0.9%), 118 lesions were GI-RADS 3 (54.9%), 86 lesions were GI-RADS 4 (40.0%) and 9 lesions were GI-RADS 5 (4.2%). Table 1 shows all GI-RADS categories with the corresponding histological results. According to the GI-RADS classification, we had 2 cases of ovarian cancer that were classified in the low-risk category 3 (Table 1), of which one was in an asymptomatic 80-year-old woman and the other was in a 42-year-old woman with menstrual irregularities.

**Table 1:** GI-RADS classification according to specific histopathologic diagnoses

Histopathology	GI-RADS				N	%	% of malignant	% benign
	2	3	4	5				
adenocarcinoma ovary	0	2	36	6	44	20.5%	83.0%	
carcinoma papillare	0	0	0	1	1	0.5%	1.9%	
cystadenofibroma serosum proliferans	0	0	1	1	2	0.9%	3.8%	
cystadenoma mucinosum proliferans	0	0	1	0	1	0.5%	1.9%	
cystadenoma proliferans	0	1	3	1	5	2.3%	9.4%	
corpus luteum	1	1	0	0	2	0.9%		1.2%
corpus luteum hemorrhagicum	0	1	1	0	2	0.9%		1.2%
cystadenofibroma	1	1	5	0	7	3.3%		4.3%
cystadenofibroma mucinosum	0	1	2	0	3	1.4%		1.9%
cystadenofibroma serosum	0	4	2	0	6	2.8%		3.7%
cystadenoma mucinosum	0	4	1	0	5	2.3%		3.1%
cystadenoma serosum	0	2	0	0	2	0.9%		1.2%
cystis benigna	0	0	1	0	1	0.5%		0.6%
cystis follicularis	0	2	1	0	3	1.4%		1.9%
cystis lueinisans	0	2	0	0	2	0.9%		1.2%
cystis serosa	0	16	6	0	22	10.2%		13.6%
cystis serosa paraovarialis	0	11	0	0	11	5.1%		6.8%
cystis serosa paraoviducti	0	1	0	0	1	0.5%		0.6%
cystis serosum	0	1	0	0	1	05%		0.6%
cystis simplex	0	3	0	0	3	1.4%		1.9%
dermoidalna	0	2	0	0	2	0.9%		1.2%
endometrioma	0	35	7	0	42	19.5%		25.9%
fibrothecoma	0	1	6	0	7	3.3%		4.3%
folliculoma	0	0	2	0	2	0.9%		1.2%
hydrosalpinx	0	1	1	0	2	0.9%		1.2%
myoma pedunculated	0	1	0	0	1	0.5%		0.6%
ovarian abscess	0	0	1	0	1	0.5%		0.6%
teratoma	0	25	9	0	34	15.8%		21.0%
All	2	118	86	9	215	100.0%	100.0%	100.0%

*N – number of cases*

For GI-RADS classifications 4 and 5, the sensitivity, specificity, PPV, NPV, ACC and OR values were as follows: 94.3, 72.2, 52.6, 97.5, 77.7% and 43.3, respectively (CI 12.0–146). For the GI-RADS classification combined with the CA-125 marker, the sensitivity, specificity, PPV, NPV, ACC and OR values were as follows: 66.0, 93.8, 77.8, 89.4, 87.0% and 29.6, respectively (CI 12.6–69.6, *p* < 0.001). For Ca-125 > 30 IU/mL alone, the sensitivity, specificity, PPV, NPV, ACC and OR values were as follows: 70.0, 80.3, 53.8, 89.1, 77.7% and 9.5, respectively (CI 4.6–19.6, *p* < 0.0001) (Table 2).

**Table 2:** Statistical analysis of GI-RADS classification and levels of the ovarian malignancy marker CA-125

Descriptive Statistics	Sensitivity	Specificity	PPV	NPV	ACC	OR	OR 95% CI	*p*-value
GI-RADS + CA 125 > 30 IU/mL	66.0%	93.8%	77.8%	89.4%	87.0%	29.6	12.6–69.6	< 0.00001
GI-RADS 4–5	94.3%	72.2%	52.6%	97.5%	77.7%	43.3	12.9–14.6	< 0.0000
CA-125 > 30 IU/mL	70.0%	80.3%	53.8%	89.1%	77.7%	9.5	4.6–19.6	< 0.0000

*IU/ml* international units per milliliter, *ACC* accuracy, *PPV* positive predictive value, *NPV* negative predictive value, *OR* odds ratio, *CI* confidence interval

GI-RADS classification had the highest sensitivity of all methods used. The application of Ca-125 measurement as an additional differentiation criterion improved the specificity of GI-RADS: 93.8% (with CA-125) vs 72.2% (without). Other descriptive statistics also seemed to have improved as well: a PPV of 77.8% vs 52.6% and an accuracy of 87% vs 77.7% with and without Ca-125, respectively. Unfortunately, the odds ratio decreased by approximately 30%, from 43.3 to 29.6. However, the odds ratio was still considerably higher for the combined measure than for Ca-125 alone: 29.6 vs 9.5.

The percentage of malignant tumors in our study was quite high (24.7%). The most frequent histological manifestation was adenocarcinoma (44 cases), which constituted approximately 83% of all the malignant cases. There were two malignant tumors classified as GI-RADS 3 (“probably benign”), which comprised 3.77% of all malignant cases. We classified a total of 42 lesions as “probably malignant” or “very probably malignant”, which corresponded to 36 cases of GI-RADS 4 (85.7%) and 6 cases of GI-RADS 5 (13.6%), respectively. Among the malignant ovarian tumors, we diagnosed 9 cases (20.5% of the malignant cases, and 3.7% of the adnexal masses).

Regarding symptoms, 47.8% of patients were symptom-free and the rest had back pain (36.5%), increased abdominal size (5.2%), menstrual irregularities (4.3%) and constipation (2.6%) (Table 3). In the low-risk group (GIRADS 1–3 and GIRADS 4 with CA-125 < 30 IU/mL), we report 152 benign and 18 malignant cases. In the high-risk group (GIRADS 4 with CA-125 > 30 IU/mL and GIRADS 5), we report 10 benign and 35 malignant tumors (Table 4).

**Table 3:** Clinical symptoms of women with adnexal masses

Clinical symptoms	GI-RADS				N	%
	2	3	4	5		
Lack of symptoms	1	64	44	1	110	47.8%
Back pain	1	50	32	1	84	36.5%
Increased abdomen size	1	0	6	5	12	5.2%
Menstrual irregularities^a^	0	3	5	2	10	4.3%
Constipation	0	2	3	1	6	2.6%
Weight loss	0	0	2	0	2	0.9%
Pain during intercourse^b^	0	2	0	0	2	0.9%
Nausea	0	1	0	0	1	0.4%
Leg swelling	0	0	1	0	1	0.4%
Frequent urination	0	0	1	0	1	0.4%
Urinary retention	0	0	1	0	1	0.4%
All	3	122	95	10	230	100.0%

^a^only for premenopausal women, ^b^for sexually active women; *N –* number of cases

**Table 4:** Diagnostic performance of GI-RADS classification with CA-125

GI-RADS	Benign	Malignant
GI-RADS 1–3 and GI-RADS 4 + CA-125 < 30 IU/mL	152	18
GI-RADS 4 + CA-125 > 30 IU/mL and GI-RADS 5	10	35
All	162	53

## Discussion

We found that using GI-RADS classification is not an effective method for predicting the malignancy of ovarian tumors when combined with CA-125 level measurement. When the GI-RADS system is combined with CA-125 levels of > 30 IU/ml, we report low sensitivity and high specificity for malignancy discrimination (66.0 and 93.8%, respectively). We also found that for GI-RADS 4 and 5, GI-RADS had higher sensitivity but lower specificity than for lower GI-RADS classifications: 94.3 and 72.2%, respectively. The results regarding GI-RADS performance are similar to those published by Zhang et al., despite the fact that the authors did not analyze the CA-125 levels as an additional marker for malignancy discrimination [12]. Following Amor et al., we support the statement that the GI-RADS classification system is useful for clinical decision-making and patient management [11, 13]. Due to the progress in the image quality and resolution of transvaginal ultrasound, image scores improve the objectivity and accuracy of ovarian tumor diagnosis [13]. Furthermore, ovarian tumor morphology assessment is subjective and requires the training and experience of sonographers to maintain a high quality of performance [14]. GI-RADS classification was developed in 2009 to simplify communication between sonographers and clinicians/gynecologists [11]. It is suggested that GI-RADS 4 and 5 cases be referred to a gynecological oncologist due to the 20% risk of malignancy [13]. Moszynski et al. highlight that the GI-RADS classification is a subjective measure, especially in the case of tumors classified as GI-RADS 4, which are considered to be difficult to assess [14]. Although there are other methods and scoring systems to distinguish between malignant and benign ovarian tumors, these methods have complex scoring and regression of ultrasonographic findings and require combining the ultrasonographic results with laboratory indexes [10, 12, 15, 16]. More data is needed, however, for GI-RADS classification performance when used by nonexpert examiners.

The assessment of biomarkers may be a more objective method suitable for less-experienced ultrasonographers [14]. CA-125 is the most popular and widely used ovarian cancer marker, but its effectiveness in terms of ovarian cancer differential diagnosis is questionable [1, 4, 17–21]. While CA-125 is quite accurate among postmenopausal women, its many false-positive results in premenopausal patients are a main limitation [22]. Our cutoff value for CA-125 levels was 30 IU/ml, which can explain the low sensitivity (70%). Niemi et al. report a CA-125 sensitivity of 59.4% with a cutoff of 35 kU/ml, whereas Wang et al., using the same cutoff value, report a sensitivity and specificity of 85.9 and 85.2%, respectively [18]. The main reason for the late-stage increase in the CA-125 serum concentration could be the molecular weight of the protein, 200–1000 kDa, compared to that of human epididymis protein 4 (HE4), which is 25 kDa. The other clinical implication is the lack of specificity of CA-125 in patients with endometriosis. Thus, it is easy to misdiagnose ovarian endometriosis as ovarian cancer, which can lead to significant physical and physiological harm inflicted to patients [23]. Koneczny et al. report that the IOTA group LR1 and GI-RADS performed well when used by either experienced or less-experienced operators of ultrasound systems [24]. For prognostic models such as GI-RADS, very high sensitivity (94.6%) and good specificity (75.5%) for examiners at level III and level II (72.7 and 87.8%, respectively) was reported. Nevertheless, in our study, we report that combining GI-RADS with CA-125 measurements can yield improved values for diagnostic parameters such as sensitivity, specificity, PPV, NPV, ACC and OR, which were 66.0, 93.8, 77.8, 89.4, 87.0% and 29.6, respectively. A study by Lycke et al. reported that in postmenopausal women, RMI (> 200), ROMA (>/=29.9), CA-125 (> 35 U/ml), and HE4 (> 140 pmol/l) showed a sensitivity of 89, 91, 92, and 72% and a specificity of 80, 77, 80, and 92%, respectively. In premenopausal women, the sensitivity of RMI, ROMA (>/=11.6), CA125, and HE4 (> 70 pmol/l) was 87, 87, 96, and 83%, and the specificity was 90, 81, 60, and 91% [25], respectively. These results suggest that CA125 is superior to HE4 as a biomarker to identify women with ovarian cancer. HE4 is better at identifying benign lesions, which may help with differential diagnoses to guide the level of care and decrease overtreatment [25].

In evaluating the symptoms, we noticed that 47.8% of all cases were actually symptom-free. If present, symptoms were nonspecific, such as back pain (36.5%), while an increased abdominal size was typical for GI-RADS 4 and 5 cases. Pitta et al. reported good discrimination of tumors based on the Ward agglomerative method for hierarchical clustering using the following symptoms: abdominal bloating and/or increased abdominal size, back pain, leg swelling, eating (unable to eat, feeling full quickly), feeling of abdominal mass, miscellaneous (fatigue and or difficulty breathing), digestion (indigestion and/or nausea/vomiting), bladder (urinary urge and/or frequent urination), and in combination with CA-125, this guidance should facilitate decision making for primary care physicians [4, 26]. In our opinion, this promising data presented by Pitta et al. enhanced further prospective research.

Our study has some limitations. First, this study was a retrospective study. Second, this study was based on data from only one health center, yielding a rather small cohort and possible examiner bias. Third, in our study, we had 2 ovarian cancers classified as low-risk (GI-RADS 3), of which one case was in a symptomless 80-year-old patient and the other case was in a 42-year-old patient with menstrual irregularities.

In conclusion, the GI-RADS classification showed good performance in discriminating ovarian tumors. GI-RADS is considered a useful tool for the management of patients with an adnexal mass who are referred to a tertiary center. When combined with the measurement of CA-125, the test specificity, PPV and ACC for the assessment of preoperative adnexal tumors is improved. Future studies should seek clinically sensitive imaging diagnostic methods for ovarian pathologies to establish an integrated, relatively specific system for early warning of tumors.

## Abbreviations

CA-125: cancer antigen 125
GI-RADS: Gynecology imaging reporting and data system
